# The Transient Multidrug Resistance Phenotype of *Salmonella enterica* Swarming Cells Is Abolished by Sub-inhibitory Concentrations of Antimicrobial Compounds

**DOI:** 10.3389/fmicb.2017.01360

**Published:** 2017-07-19

**Authors:** Oihane Irazoki, Susana Campoy, Jordi Barbé

**Affiliations:** Departament de Genètica i de Microbiologia, Universitat Autònoma de Barcelona Barcelona, Spain

**Keywords:** swarming, transient multidrug-resistance phenotype, SOS response, chemoreceptor polar arrays, cell flagellation

## Abstract

Swarming motility is the rapid and coordinated multicellular migration of bacteria across a moist surface. During swarming, bacterial cells exhibit increased resistance to multiple antibiotics, a phenomenon described as adaptive or transient resistance. In this study, we demonstrate that sub-inhibitory concentrations of cefotaxime, ciprofloxacin, trimethoprim, or chloramphenicol, but not that of amikacin, colistin, kanamycin or tetracycline, impair *Salmonella enterica* swarming. Chloramphenicol-treated *S. enterica* cells exhibited a clear decrease in their flagellar content, while treatment with other antibiotics that reduced swarming (cefotaxime, ciprofloxacin, and trimethoprim) inhibited polar chemoreceptor array assembly. Moreover, the increased resistance phenotype acquired by swarming cells was abolished by the presence of these antimicrobials. The same occurred in cells treated with these antimicrobial agents in combination with others that had no effect on swarming motility. Our results reveal the potential of inhibiting swarming ability to enhance the therapeutic effectiveness of antimicrobial agents.

## Introduction

Swarming motility is the rapid and coordinated multicellular migration of bacteria across a moist surface mediated by flagella (Henrichsen, 1972). This motility is widely distributed throughout flagellated bacteria and is associated with their colonization of host surfaces, the increased expression of virulence factors, and antibiotic resistance (Kim and Surette, 2003, 2004; Overhage et al., 2008; Lai et al., 2009). Specifically, bacterial colonies exhibit a greater resistance to multiple antibiotics when swarming (Kim and Surette, 2003; Kim et al., 2003; Lai et al., 2009; Butler et al., 2010). This adaptive antibiotic resistance has been described for temperate swarmers such as *Salmonella enterica, Escherichia coli*, and *Pseudomonas aeruginosa*. In these species, swarming-associated resistance is non-genetically conferred, since it ceases when the cells are grown under non-swarming conditions (Overhage et al., 2008; Lai et al., 2009; Butler et al., 2010). The mechanism underlying this transient resistance is poorly understood but it may be a physiological attribute of swarming cells, related, for example, to an altered outer membrane composition (Kim and Surette, 2003) or a decrease in membrane permeability (Kim and Surette, 2004) acquired in response to bacterial growth on moist surfaces. Furthermore, multidrug-resistance seems to be a function of the bacterial cell density coupled with the swarming velocity of the bacterial colony (Butler et al., 2010).

*Salmonella enterica* cells adapt their surface motility in response to the presence of direct or indirect DNA-damaging agents by sensing these compounds through the so-called SOS response (Irazoki et al., 2016b). Among the consequences of SOS system activation is an increase in RecA protein concentration within the cells. RecA is both the main bacterial recombinase and the DNA-damage sensor of the SOS system (Little et al., 1980; Cox, 2008). An increase in RecA during the SOS response leads to an impaired swarming ability, via the titration of the CheW protein (Irazoki et al., 2016a,b).

The CheW protein, together with other components of the chemotaxis pathway, plays a key role in swarming ability (Burkart et al., 1998; Mariconda et al., 2006). As the chemoreceptor adaptor, CheW couples transmembrane methyl-accepting chemoreceptor protein (MCP) trimers of dimers to the histidine kinase CheA (Boukhvalova et al., 2002; Sourjik and Wingreen, 2012). The signal recognition at the chemoreceptor level generates conformational changes that modulate the CheA autophosphorylation activity. This signal is transmitted through a phosphorylation cascade to CheY (CheY∼P), the response regulator that modulates the flagellar motor rotation. To avoid saturation of the sensory system, the chemoreceptor signal is reset by the activity of a methyltransferase (CheR) and a methylesterase (CheB). Both proteins are located in the vicinity of the chemoreceptors to restore pre-stimulus activity through reversible covalent methylation of the MCPs (Sourjik and Wingreen, 2012). Stabilized by CheW and CheA hexagonal rings, these signaling complexes aggregate at the cell poles to form the large chemoreceptor arrays that are essential for the surface motility of temperate swarmers (Zhang et al., 2007; Cardozo et al., 2010; Santos et al., 2014). An increase in intracellular RecA levels due to SOS response activation hijacks CheW, thus preventing stabilization of the chemoreceptor cluster at the cell poles and impairing swarming motility (Irazoki et al., 2016a,b).

Increases in bacterial resistance to antimicrobials have compromised the clinical utility of major chemotherapeutic antimicrobial agents. Other factors compromising the efficacy of these drugs are the administration of sub-optimal doses and poor pharmacokinetics, due, for example, to inefficient tissue penetration. To explore the possible inhibitory effect of antimicrobial compounds on both swarming motility and the transient acquisition of multidrug resistance, we analyzed the swarming ability and antibiotic resistance phenotype of *S. enterica* in experiments conducted using sub-inhibitory concentrations of several antimicrobial compounds differing in their mechanisms of action. Furthermore, as not only the functional chemotaxis system but the presence of polar chemosignaling arrays is essential for swarming motility in temperate swarmers, we also examined the chemoreceptor array assembly and flagellation of antibiotic-treated cells. Our results demonstrate that some antimicrobial agents, alone or in combination with others not affecting cell motility, prompt not only swarming inhibition but also the abolishment of transient multidrug resistance.

## Materials and Methods

### Bacterial Strains and Growth Conditions

*Salmonella enterica* sv. Typhimurium ATCC14028 wild-type and Δ*recA* strains (Medina-Ruiz et al., 2010) and their Δ*cheR* mutant derivatives carrying plasmid pUA1127, harboring an *eYFP::cheR* fusion (Mayola et al., 2014), were used in this work.

Except when indicated, the cells were grown at 37°C on either Luria–Bertani (LB) plates containing 1.7% agar or on swarming plates (1% tryptone, 0.5% yeast extract, 0.5% NaCl, 0.5% D-(+)-glucose, and 0.5% agar). These conditions are referred to in the following as non-swarming and swarming conditions, respectively.

When necessary, the plate media were supplemented with a sub-inhibitory concentration of amikacin (4 mg/L), cefotaxime (1.6 mg/L), chloramphenicol (2 mg/L), colistin (2.5 mg/L), tetracycline (4 mg/L), kanamycin (5 mg/L), ciprofloxacin (0.0065 mg/L), and/or trimethoprim (1 mg/L). In all cases, antibiotics were filtered and the corresponding antimicrobial was added to the media after the sterilization process when cooled down.

### MIC and Sub-inhibitory Concentration Determination

The minimal inhibitory concentrations (MICs) of the antibiotics used in this work for *S. enterica* ATCC14028 and the Δ*cheR* derivative with pUA1127 plasmid containing the *eYFP::cheR* fusion were determined by the standard microdilution method using tryptone broth (TB), as described previously (Wiegand et al., 2008). The obtained values for both strains were similar. Strain growth ability was tested in microtiter plate wells containing twofold serial dilutions of the antibiotic in TB (Supplementary Table S1). The sub-inhibitory concentration was defined as the antimicrobial concentration inhibiting growth by 20–30% compared to non-treated cells. Bacterial growth reduction was determined by measuring the optical density of bacterial cultures at 600 nm (Supplementary Figure S1) as described previously (Kohanski et al., 2010). Further, in all cases, it was confirmed that the established sub-inhibitory concentration reduced *S. enterica* viability by ∼30% after 2 h of treatment (Supplementary Table S2).

### Swarming Motility Assays

The swarming phenotype of wild-type *S. enterica* or its Δ*cheR* derivative was tested in the presence of the above-listed antimicrobial agents. In all cases, the observed swarming phenotype of the two strains was exactly the same. When needed, the *S. enterica* Δ*recA* strain, which is unable to swarm (Medina-Ruiz et al., 2010), was also used as a non-motile control.

Swarming assays were carried out as described previously (Gómez-Gómez et al., 2007; Mayola et al., 2014; Irazoki et al., 2016b). Briefly, a single colony picked using a sterile toothpick from bacterial strains grown on LB plates was inoculated in the center of a freshly prepared swarming plate containing medium supplemented with the antimicrobial compound(s) of interest. The plates were incubated overnight at 37°C for 14 h and then imaged using a ChemiDoc^TM^ XRS+ system (Bio-Rad).

### Chemoreceptor Clustering Assay

Chemoreceptor clustering assays were performed using *S. enterica* Δ*cheR* carrying plasmid pUA1127 containing the *eYFP::cheR* fusion under the control of an IPTG-inducible promoter (Ptac) (Mayola et al., 2014). The fusion protein served as a reporter of polar cluster localization (Kentner et al., 2006; Cardozo et al., 2010; Santos et al., 2014). The clustering experiments were carried out as described previously (Mayola et al., 2014; Irazoki et al., 2016b). Briefly, overnight cultures were grown under constant agitation at 30°C in TB supplemented with ampicillin and 25 μM IPTG. The day after, the cultures were diluted 1:100 in TB without antibiotics but with the addition of 25 μM IPTG to maintain the induction of the *eYFP::cheR* fusion construct. The cultures were incubated at 30°C until an OD_600_ of 0.08–0.1 was reached, when the appropriate antimicrobial was added to each culture. Samples were collected at the indicated times and the cells were harvested by low-speed centrifugation for 15 min. The harvested cells were washed once using ice-cold tethering buffer (10 mM potassium-phosphate pH 7, 67 mM NaCl, 10 mM Na-lactate, 0.1 mM EDTA, and 0.001 mM L-methionine), resuspended in 20–100 μL of tethering buffer, and applied onto thin 1% agarose pads.

Fluorescence microscopy was performed using a Zeiss Axio Imager M2 microscope (Carl Zeiss Microscopy) equipped with a Zeiss AxioCam MRm monochrome camera (Carl Zeiss Microscopy) and a filter set for enhanced yellow fluorescent protein (eYFP; excitation BP500/25; beam splitter FT 515; emission BP535/30). Cell fields were photographed and the number of clusters then quantified using ImageJ software (National Institutes of Health). At least 500 cells were visually inspected. Each experiment was performed at least in triplicate using independent cultures, resulting in the examination of a minimum of 1500 cells from each growth condition.

### ELISA for RecA Quantification

Samples for RecA quantification were obtained by recovering the cells directly from the colony edge of the corresponding swarming plates, following the same procedure and conditions as described above. In this case, the cells were resuspended in sonication buffer (PBS 1×, cOmplete mini EDTA-free tablets, pH 7.3) and whole-cell lysates were obtained by sonication (two 30-s pulses of 20% amplitude, Digital sonifier^®^ 450, Branson). The supernatants were recovered, centrifuged (12000 *g* for 10 min), and the total protein concentration of each sample was then quantified using the Bradford method (protein reagent DyeR, BioRad). A standard curve was generated using bovine serum albumin (range: 1.5–200 μg/mL).

Pre-treated 96-well microtiter plates (Nunc-Immunoplate F96 Maxisorp, Nunc) were coated with serial dilutions of the whole-cell lysates. The RecA concentration in the polar cluster assays was determined by ELISA, as previously described (Irazoki et al., 2016b). A standard quantification curve was obtained using purified RecA protein. A rabbit anti-RecA antibody (ab63797, Abcam) served as the primary antibody, and a goat anti-rabbit-IgG horseradish-peroxidase-conjugated antibody (IgG, IgM, IgA, polyclonal antibody, YO Proteins) as the secondary antibody. The BD OptEIA TMB substrate reagent kit (BD Biosciences) was used as the developing solution. The plate reactions were read at 650 nm using a multi-plate reader (Sunrise, Tecan).

### Disk-Diffusion Sensitivity Test

To evaluate the antibiotic susceptibilities of the cells, LB non-swarming or swarming plates supplemented when needed with the corresponding antibiotic were surface-inoculated using a sterile swab with either *S. enterica* Δ*cheR* pUA1127 or *S. enterica* Δ*cheR* Δ*recA* pUA1127 freshly grown on LB plates. Antimicrobial susceptibility test disks (amikacin, 30 μg; trimethoprim, 25 μg; and tetracycline, 30 μg; Pronadisa) were placed in the middle of the inoculated plates. After a 14 h incubation at 37°C, the size of the bacterial growth inhibition zone was determined based on photographic images of the plates (ChemiDoc XRS + system, Bio-Rad). Each of these experiments was performed at least in triplicate. The images shown in the figures are representative of the entire image set.

### Fluorescence Flagellar Labeling

The flagella were labeled as described previously (Turner et al., 2010) but with modifications. Cells from the corresponding swarming plates were collected in 0.5 mL of tethering buffer (described above), washed three times by centrifugation (1500 ×*g* for 10 min) at 15°C, and resuspended first in 1 mL and then in 0.5 mL of PBS. To label the flagella, 100 μL of PBS, 25 μL of 1 M NaHCO_3_, and 0.5 μL of Cy3b dye (0.1 mg/μL) were added to the cell suspension. After a 1-h incubation at room temperature in the dark with gyrorotation, the labeled cells were washed three times with 1 mL of PBS before they were resuspended in 0.2–0.5 mL of tethering buffer.

To visualize the *S. enterica* ATCC14028 cells, they were immobilized and fixed on the same focal plane using thin 1% agarose pads in tethering buffer. Fluorescence microscopy was performed using a Zeiss Axio Imager M2 microscope (Carl Zeiss Microscopy) equipped with a Zeiss AxioCam MRm monochrome camera (Carl Zeiss Microscopy) and a filter set for Cy3b protein (excitation BP 546/12; beam splitter FT 560; emission BP 575-640). All fluorescence images were obtained at a 100× magnification under identical conditions. Each experiment was performed in triplicate using independent cultures. The images presented are representative of the entire image set. ImageJ software (National Institutes of Health) was used to prepare images for publication. In all cases, at least 100 cells were visually inspected. Each experiment was performed at least in triplicate using independent cultures, resulting in the examination of a minimum of 300 cells from each growth condition.

### Statistical Analysis

Data from the polar clustering assays and measurements of the RecA concentration were analyzed using a one-way analysis of the variance (ANOVA) with Prism (GraphPad). In all cases, the analyses were followed by Bonferroni multiple comparison *post hoc* tests. A *p*-value < 0.01 was considered to indicate statistical significance. The error bars in each of the figures indicate the standard deviation.

## Results

### Swarming Ability in the Presence of Sub-inhibitory Concentrations of Antimicrobial Agents

**FIGURE 1:**
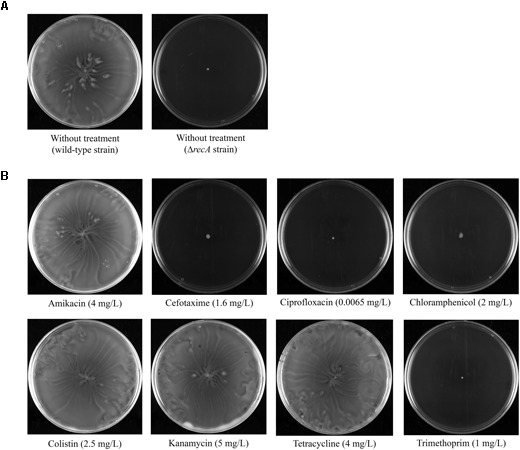
**(A)** Swarming ability of *S. enterica* wild-type and a Δ*recA* mutant derivative grown on swarming-plates. **(B)** Effect of sub-inhibitory concentrations (indicated in parentheses) of several antimicrobial agents on the swarming ability of the *S. enterica* wild-type strain. Representative images of a swarming bacterial colony are shown.

The effect on swarming of sub-inhibitory concentrations of the following antimicrobial agents differing in their mechanisms of action was analyzed: (i) inhibitors of translation (chloramphenicol, tetracycline, and the aminoglycosides kanamycin and amikacin); (ii) an inhibitor of cell-wall synthesis (the cephalosporin cefotaxime); (iii) an inhibitor of DNA replication (the fluoroquinolone ciprofloxacin); (iv) a disruptor of the outer cell membrane (colistin); and (v) an inhibitor of thymidine synthesis (trimethoprim). Swarming plates containing sub-inhibitory concentrations of the corresponding antimicrobial agent were inoculated with *S. enterica* wild-type strain in the middle of the plate. In all cases, it was determined that the antimicrobial concentration used produces about 30% reduction in cell viability when grown in liquid media (see Materials and Methods). In addition, the swarming ability of the wild-type strain and a Δ*recA* mutant derivative, which is not able to swarm (Medina-Ruiz et al., 2010), were tested in the absence of any antimicrobial agent, as swarming and non-swarming controls, respectively (**Figure 1A**).

Our results indicated that the presence of kanamycin, amikacin, colistin, and tetracycline did not affect the swarming ability of *S. enterica* (**Figure 1B**); rather, the phenotype of the respective bacterial colonies was the same as that of wild-type non-treated cells (**Figure 1A**). By contrast, the addition of sub-inhibitory concentrations of cefotaxime, ciprofloxacin, trimethoprim, and chloramphenicol completely abolished swarming motility (**Figure 1B**). In these cases, the sub-inhibitory concentration of antimicrobial treatment gave rise to the same non-swarming phenotype as observed for non-treated Δ*recA* cells (**Figure 1A**).

### Identification of the Antibiotic Mediated Swarming Abolition Mechanism

To identify the mechanism responsible for impaired swarming, the dynamics of chemosensory array assembly and the flagellation of swarming cells during antibiotic treatment were evaluated, since both are essential for the surface motility of *S. enterica* (Cardozo et al., 2010; Partridge and Harshey, 2013; Santos et al., 2014). The polar clustering assays were performed using cells grown in liquid media, since it has been previously described that the number of cells presenting polar clusters is similar regardless of whether they are grown in liquid or on swarming plates (Irazoki et al., 2016b).

**FIGURE 2:**
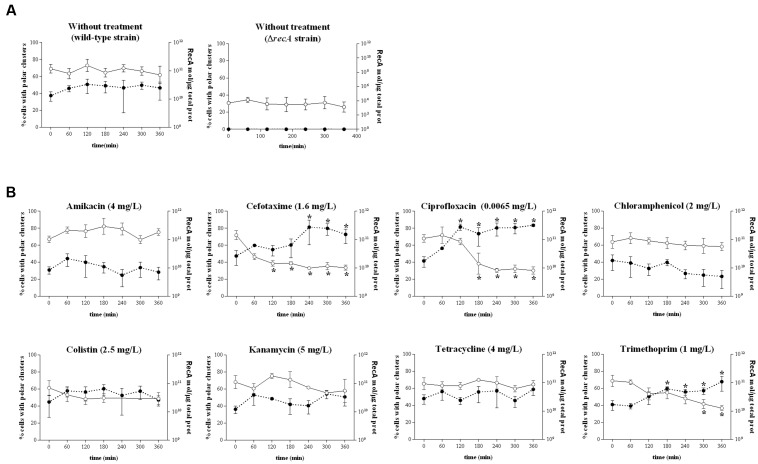
Evolution of the polar chemoreceptor cluster assembly (expressed as a percentage of the entire population) of the *S. enterica* Δ*cheR*/pUA1127 strain expressing the *eYFP::cheR* fusion grown in the absence **(A)** or presence **(B)** of sub-inhibitory concentrations of the indicated antibiotics. Cluster assembly was measured at several time points after the addition of each antibiotic (white dots, continuous line). The antibiotic concentration is indicated in parentheses. The RecA concentration in each sample, quantified by ELISA, is also shown (black dots, discontinuous line). *S. enterica* Δ*recA* cells cultured in the absence of antibiotic were included as a non-swarming strain control. In all cases, the results are the mean of at least three independent imaging, swarming, or ELISA experiments. Error bars indicate the standard deviation. ^∗^*p* < 0.01 compared to the initial sample.

Chemosensory arrays were observed using a *S. enterica* strain expressing the *eYFP::cheR* fusion under the control of an IPTG-inducible promoter (Supplementary Figure S2; Mayola et al., 2014; Irazoki et al., 2016b). This strain exhibits the same swarming phenotype as the wild-type strain. In concordance with their motility behavior, cells treated with the antimicrobials that allowed swarming (kanamycin, amikacin, tetracycline, and colistin) did not exhibit altered polar chemosensory array assembly (**Figure 2B**) compared to non-treated cells (**Figure 2A**). Consistent with this finding, there was no alteration in the RecA concentration in cells treated with the compounds that did not impair surface motility (**Figure 2B**).

However, among the agents that blocked swarming, treatment with cefotaxime, ciprofloxacin, and trimethoprim but not chloramphenicol induced a significant decrease in polar cluster assembly (**Figure 2B** and Supplementary Figure S2). Furthermore, the increase in the intracellular concentration of RecA mediated by cefotaxime, ciprofloxacin, and trimethoprim indicates the induction of the SOS response by these antibiotics (**Figure 2B**). The percentage of cells with polar clusters at the end of the corresponding treatment was similar to that observed in the Δ*recA* strain (**Figure 2A**). Moreover, and as expected, when swarming cells were treated with antibiotic concentrations of cefotaxime, ciprofloxacin, and trimethoprim that did not induce the SOS response no swarming impairment was observed (Supplementary Figure S3). Previous reports showed that either the increase in the RecA concentration following SOS response induction by mitomycin-C treatment or the absence of this protein within Δ*recA* mutant cells prevents polar chemosensory array formation due to CheW-RecA titration (Mayola et al., 2014; Irazoki et al., 2016a).

**FIGURE 3:**
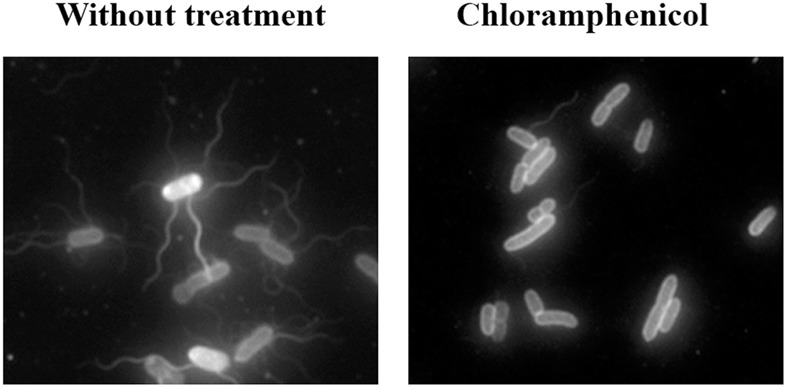
Representative fluorescence microscopy images of Cy3b-labeled *S. enterica* wild-type grown on swarming plates in the presence or absence of chloramphenicol (2 mg/L).

To visualize the flagella, the cells were directly labeled with Cy3b fluorescent dye, as previously described (Turner et al., 2010). As expected, treatment with the antimicrobial compounds that allowed swarming had no effect on cell flagellation, which was the same as in non-treated cells (data not shown). However, despite the inhibitory effect of cefotaxime, ciprofloxacin, and trimethoprim on swarming, cells treated with these compounds had the same flagellar phenotype as non-treated bacteria (data not shown). Only in cells treated with a sub-inhibitory concentration of chloramphenicol was there a clear reduction in the number of flagella (**Figure 3**), which explained the abolishment of swarming by this antibiotic.

### Drug-Resistance Phenotype of Swarming-Impaired Cells

**FIGURE 4:**
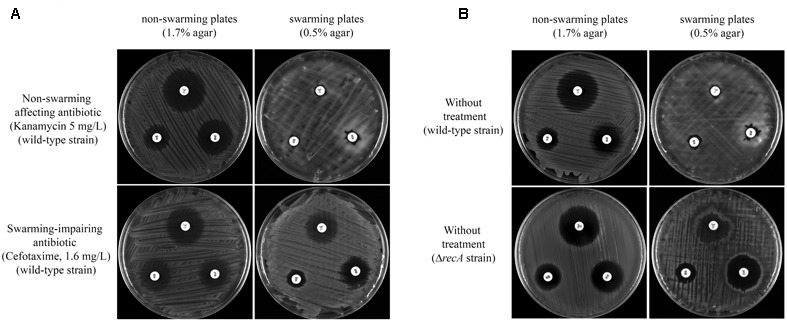
**(A)** Multidrug resistance phenotype of *S. enterica* Δ*cheR*/pUA1127 cells grown on swarming and non-swarming plates in the presence of sub-inhibitory concentrations of antibiotics that either inhibit (cefotaxime) or have no effect on swarming (kanamycin). Disk-diffusion sensitivity tests were used to determine the sensitivity to trimethoprim (upper disk), amikacin (left disk), and tetracycline (right disk) of *S. enterica* Δ*cheR*/pUA1127 cells grown on plates containing 1.7% (non-swarming plates) or 0.5% (swarming plates) agar supplemented with the corresponding antibiotic. **(B)** As controls, the same disk-diffusion sensitivity tests were performed using *S. enterica* Δ*cheR*/pUA1127 and Δ*recA* mutant derivative grown on plates lacking supplemented antimicrobial agents.

*S. enterica* swarming cells exhibit elevated resistance to a variety of antibiotics, including those that target the cell envelope, protein translation, DNA replication, and transcription (Kim and Surette, 2003; Kim et al., 2003; Butler et al., 2010). To determine whether the antibiotics that impaired surface motility also inhibited the drug increased resistant phenotype, the antibiotic susceptibility of swarming cells to trimethoprim, amikacin, and tetracycline was tested in the presence or absence of sub-inhibitory concentrations of either non-swarming affecting or swarming impairing compounds (**Figure 4A**). These three antimicrobial agents (trimethoprim, amikacin and tetracycline) were selected based on the greater level of resistance to them exhibited by cells grown on swarming plates (**Figure 4B**) as well as their different modes of action (Brogden et al., 1982; Davis, 1987; Chopra et al., 1992).

Disk-diffusion sensitivity tests revealed that the increased-resistance phenotype was preserved in the presence of a sub-inhibitory concentration of kanamycin, which did not affect swarming behavior (**Figure 4A**). In this case, kanamycin-treated cells had an increased resistance to trimethoprim, amikacin, and tetracycline when growing on swarming plates, as occurred in the absence of treatment (**Figure 4B**). The same was observed when, instead of kanamycin, sub-inhibitory concentrations of either amikacin, colistin, or tetracycline were used (data not shown). These results confirmed that compounds unable to inhibit swarming also did not affect the increased swarming-associated drug resistance phenotype.

By contrast, this increased antibiotic resistance was dramatically abolished in *S. enterica* wild-type strain treated with a sub-inhibitory concentration of cefotaxime when growing on swarming plates (**Figure 4A**). Likewise, the *recA* defective mutant, which is unable to swarm (Medina-Ruiz et al., 2010), also did not increase its resistance sensitivity to trimethoprim, amikacin, and tetracycline when growing under swarming conditions (**Figure 4B**). Furthermore, the increased drug-resistance phenotype was also inhibited by other antimicrobials that hindered swarming as ciprofloxacin, trimethoprim, and chloramphenicol (data not shown).

Together, these data indicated that the restoration of antimicrobial sensitivity is directly associated with the absence of swarming and not specifically with the inhibition of chemosensory array assembly (induced by cefotaxime, ciprofloxacin, or trimethoprim) or the reduction in the number of flagella (chloramphenicol).

### Effect of Combined Antibiotic Treatment

Combination antibiotic therapy takes advantage of possible synergistic effects between antibiotics (Tamma et al., 2012; Tängdén, 2014). For this reason, and considering the above results, we asked whether compounds that impaired swarming and hindered the increased drug-resistance phenotype maintained their effects when provided together with antimicrobials that did not alter motility. Thus, the cells were treated with sub-inhibitory concentrations of cefotaxime in combination with sub-inhibitory treatments of either kanamycin or colistin. Swarming ability, chemosensory cluster assembly, and the increased resistance phenotype were then examined.

**FIGURE 5:**
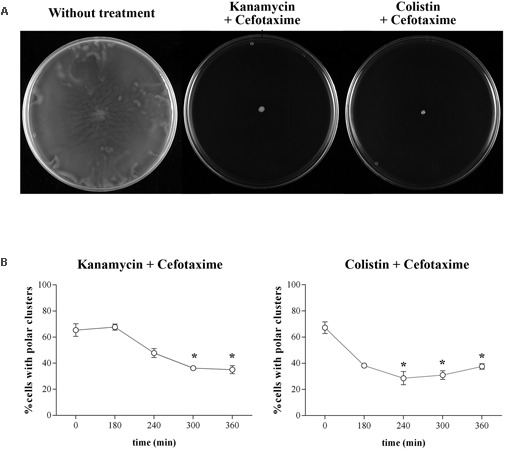
**(A)** Swarming ability of *S. enterica* Δ*cheR*/pUA1127 treated with sub-inhibitory concentrations of kanamycin (5 mg/L) or colistin (2.5 mg/L) in combination with a sub-inhibitory concentration of cefotaxime (1.6 mg/L). Representative images of a swarming bacterial colony are shown for each treatment. As a control, the swarming ability of untreated cells is included. **(B)** Evolution of polar chemosensory cluster assembly (expressed as a percentage of the entire population) in *S. enterica* cultured in the presence of sub-inhibitory concentrations of both kanamycin and cefotaxime or colistin and cefotaxime. Error bars indicate the standard deviation. ^∗^*p* < 0.01 compared to the initial sample.

**FIGURE 6:**
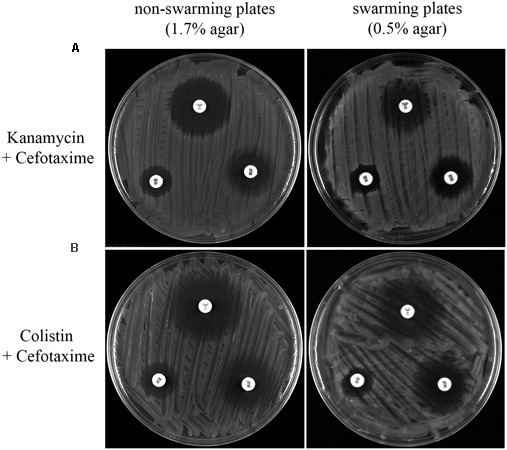
Disk-diffusion sensitivity tests comparing the trimethoprim (upper disk), amikacin (left disk), and tetracycline (right disk) resistance of *S. enterica* Δ*cheR*/pUA1127 cells grown on plates containing 1.7% (non-swarming plates) or 0.5% (swarming plates) agar supplemented with a sub-inhibitory concentration of both kanamycin and cefotaxime **(A)** or both colistin and cefotaxime **(B)**. Representative images of each condition are shown.

As shown in **Figures 5 and 6**, combined treatment with sub-inhibitory concentration of the antimicrobial agents yielded the same results as obtained with cefotaxime alone (**Figures 1, 2, 4**). Thus, in all cases, cells treated with cefotaxime and colistin or cefotaxime and kanamycin were unable to swarm (**Figure 5A**). In both cases, the number of cells with assembled polar chemosensory arrays was reduced (**Figure 5B**), and the swarming-associated increased resistance phenotype abolished (**Figure 6**). The same results were obtained when, instead of kanamycin or colistin, other non-swarming-impairing compounds, such as tetracycline or amikacin, were used in the combined treatment or when cefotaxime was replaced by other swarming-impairing compounds, such as trimethoprim or ciprofloxacin (data not shown).

Taken together these observations showed that the presence of an antibiotic with no effect on swarming (kanamycin, amikacin, colistin, or tetracycline) did not alter the effects prompted by a swarming-impairing agent (cefotaxime, ciprofloxacin, or trimethoprim).

## Discussion

The results reported herein demonstrate that sub-inhibitory concentrations of chloramphenicol, cefotaxime, ciprofloxacin, and trimethoprim abolish the swarming ability of *S. enterica* (**Figure 1B**). They also provide evidence for two different mechanisms associated with the loss of surface motility. The first is the effect of cefotaxime, ciprofloxacin, and trimethoprim, which caused a significant defect in chemosensory array assembly (**Figure 2B**). As reported in previous studies (Thi et al., 2011), sub-inhibitory concentrations of these drugs induce the SOS response, in turn prompting an increase in the RecA concentration (**Figure 2B**). It has been described that an increase of RecA within the cell impairs polar chemoreceptor array assembly by the RecA-mediated titration of CheW (Irazoki et al., 2016a,b).

The second mechanism is that promoted by chloramphenicol, which reduces the flagellation of *Salmonella* (**Figure 3**) without affecting polar chemoreceptor assembly (**Figure 2B**). In a previous report, chloramphenicol and tetracycline caused the defective motility of some *S. enterica* multidrug resistant clinical isolates, via a reduction in flagellar number (Brunelle et al., 2014). However, under the conditions of our experiments using *S. enterica* ATCC14028 swarming cells, this effect was observed in bacteria grown on plates containing a sub-inhibitory concentration of chloramphenicol but not of tetracycline, which, unlike chloramphenicol, did not abolish swarming motility (**Figure 1B**). Further work is needed to elucidate the molecular mechanisms associated with the flagellar decrease induced by chloramphenicol treatment.

The transient multidrug resistance phenotype exhibited by swarming cells is well-established (Kim and Surette, 2003; Kim et al., 2003; Butler et al., 2010). Our data are the first to demonstrate that the presence of sub-inhibitory concentration of an antimicrobial that blocks swarming (such as cefotaxime) also abolishes the increased swarming-associated drug resistance to amikacin, tetracycline, and trimethoprim of *S. enterica* cells growing on swarming plates (**Figure 4A**). These antimicrobial agents present different modes of action: amikacin binds to the 30S ribosomal sub-unit, blocking mRNA translation (Davis, 1987); tetracycline also inhibits bacterial protein synthesis, but by preventing the association of aminoacyl-tRNA with the bacterial ribosome (Chopra et al., 1992); trimethoprim exerts antimicrobial activity by blocking the production of tetrahydrofolate, the active form of folic acid (Gleckman et al., 1981). These differences indicate that the inhibition of increased drug resistance is not associated with the abolition of a specific antimicrobial resistance mechanism but with the multidrug resistance phenotype of swarming cells.

It should also be noted that the results reported herein pointed out that *S. enterica* Δ*recA*, which is unable to swarm (Medina-Ruiz et al., 2010), displays the same antibiotic susceptibility to trimethoprim, amikacin, and tetracycline when grown under swarming or non-swarming conditions (**Figure 4B**). These findings confirm that swarming ability is concomitant to increased antibiotic resistance (Kim and Surette, 2003; Kim et al., 2003; Lai et al., 2009; Butler et al., 2010).

The recent emergence of antibiotic resistance has greatly limited the therapeutic options available for treating bacterial pathogens, especially those that are multidrug-resistant. In this context, the combination therapy can offer a strategy for the treatment of severe bacterial infections if the synergistic effect of two or more antimicrobial agents in combination is greater than the sum of their individual activities (Doern, 2014). It is therefore of note that in our study the abolition of swarming-associated multidrug resistance due to the presence of a swarming-impairing compound (such as cefotaxime) was maintained even when the latter drug was used together with antimicrobials that do not affect surface motility (such as kanamycin or colistin) (**Figure 6**).

The relationship between swarming and bacterial virulence involves host surface colonization and the increased expression of virulence factors (Overhage et al., 2008; Lai et al., 2009; Medina-Ruiz et al., 2010). In fact, mutants unable to swarm are usually attenuated and often display a reduced invasiveness that can be crucial during the first steps of bacterial infection (Burall et al., 2004; Butler and Camilli, 2004; Dons et al., 2004; Stecher et al., 2004; Terry et al., 2005; Medina-Ruiz et al., 2010).

However, swarming-impairing antibiotics may also cause unwanted effects. For instance, although an increase in the RecA concentration reduces the invasiveness of *S. enterica* (Medina-Ruiz et al., 2010), activation of the SOS response by some antimicrobials increases mutagenesis and may lead to the acquisition of antibiotic resistance (Cirz and Romesberg, 2007; Petrosino et al., 2009). In the case of chloramphenicol, the induced decrease in flagellation was shown to be associated with an increase in the virulence of *S. enterica* (Brunelle et al., 2014). Therefore, the identification of compounds able to abolish swarming but not exhibiting non-desirable effects, such as the induction of SOS-mediated mutagenesis or an enhancement of virulence, would likely improve the treatment of bacterial infections.

Taken together, our study showed that compounds able to inhibit swarming motility also abolish the transient multidrug-resistant phenotype. Thus, approaches that promote the inhibition of surface motility may result in novel strategies to increase the effectiveness of antibiotic treatments targeting swarming-associated bacterial host colonization.

## Author Contributions

OI and SC analyzed swarming and multidrug resistance phenotypes and chemoreceptor polar cluster assembly. SC and JB conceived of the experiments and coordinated the research.

## Conflict of Interest Statement

The authors declare that the research was conducted in the absence of any commercial or financial relationships that could be construed as a potential conflict of interest.
