# Supplementary material for: The Transient Multidrug Resistance Phenotype of Salmonella enterica Swarming Cells Is Abolished by Sub-inhibitory Concentrations of Antimicrobial Compounds
Source: Front Microbiol. 2017 Jul 19;8:1360. doi: 10.3389/fmicb.2017.01360 (PMC5515874; doi:10.3389/fmicb.2017.01360)
Supplement: Supplementary file 1 [file Image_1.PDF]

Supplementary Material

The transient multidrug resistance phenotype of *Salmonella enterica* swarming cells is abolished by sub-lethal concentrations of antimicrobial compounds

Oihane Irazoki, Susana Campoy*, Jordi Barbé

*** Correspondence:** Corresponding Author: Susana.Campoy@uab.cat

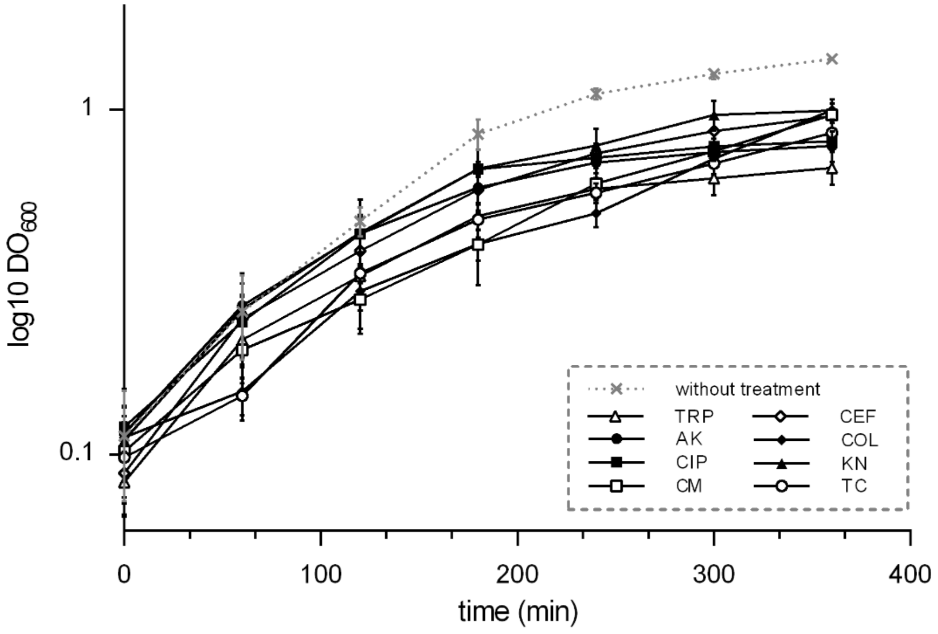

Supplementary Figure S1. Growth curves of *S. enterica* ATCC14028 *ΔcheR* pUA1127 cells growth under different antibiotic treatment. Cultures were treated with the appropriate antimicrobial dose and growth inhibition was analyzed based on OD600. A culture without treatment was included as a control.
