# Supplementary material for: The Transient Multidrug Resistance Phenotype of Salmonella enterica Swarming Cells Is Abolished by Sub-inhibitory Concentrations of Antimicrobial Compounds
Source: Front Microbiol. 2017 Jul 19;8:1360. doi: 10.3389/fmicb.2017.01360 (PMC5515874; doi:10.3389/fmicb.2017.01360)
Supplement: Supplementary file 2 [file Image_2.PDF]

frontiers

Supplementary Material

The transient multidrug resistance phenotype of *Salmonella enterica* swarming cells is abolished by sub-lethal concentrations of antimicrobial compounds

Oihane Irazoki, Susana Campoy*, Jordi Barbé

*** Correspondence:** Corresponding Author: Susana.Campoy@uab.cat

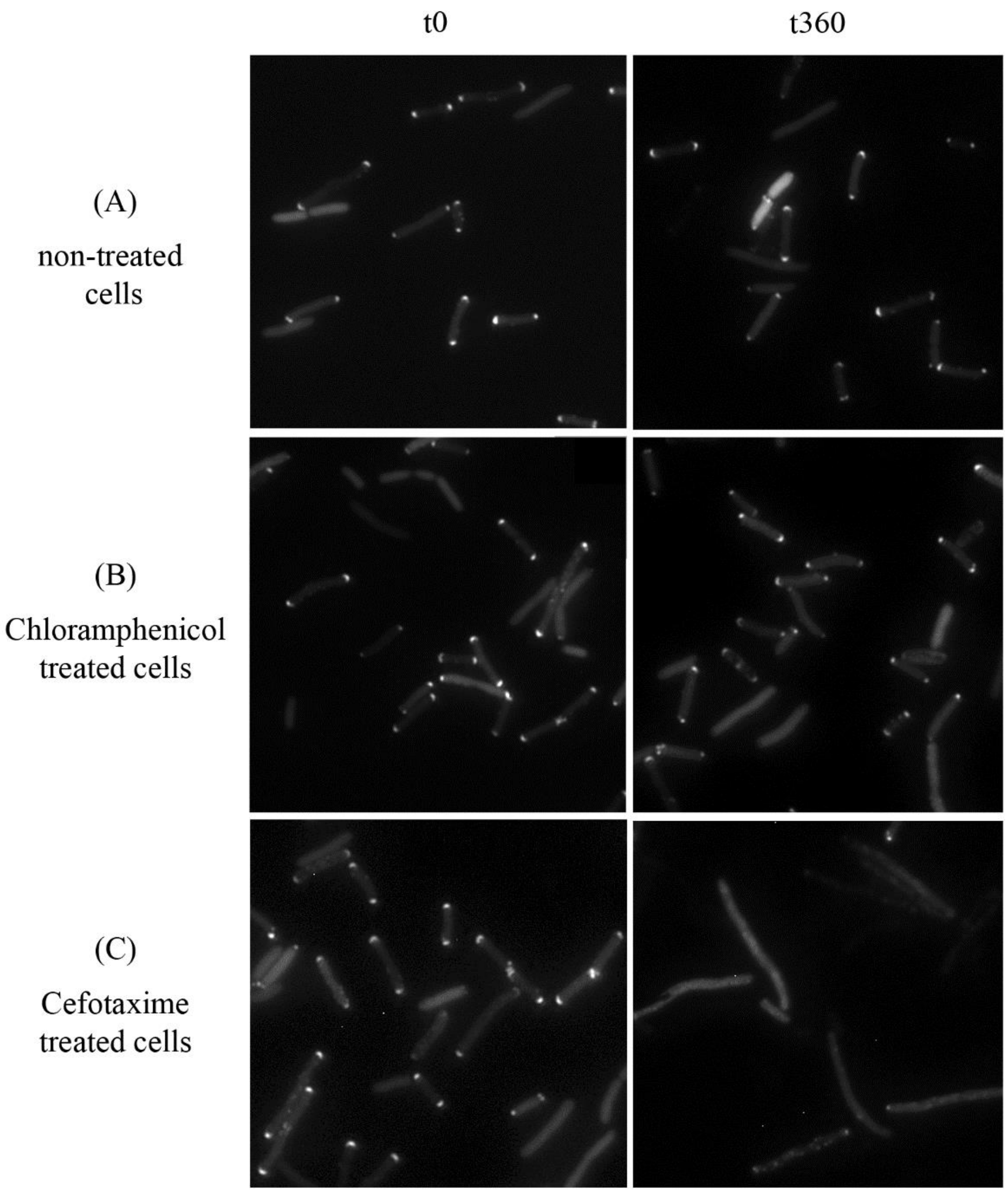

Supplementary Figure S2. Representative fluorescence microscopy images of *S. enterica ΔcheR* cells harboring plasmid pUA1127 in the absence (t0) or presence (t360) of the indicated antimicrobials. (A) non-treated cells, (B) cells treated with 2 mg/L chloramphenicol, and (C) cells treated with 1.6 mg/L of cefotaxime.
