# Supplementary material for: The Transient Multidrug Resistance Phenotype of Salmonella enterica Swarming Cells Is Abolished by Sub-inhibitory Concentrations of Antimicrobial Compounds
Source: Front Microbiol. 2017 Jul 19;8:1360. doi: 10.3389/fmicb.2017.01360 (PMC5515874; doi:10.3389/fmicb.2017.01360)
Supplement: Supplementary file 3 [file Image_3.PDF]

Supplementary Material

The transient multidrug resistance phenotype of *Salmonella enterica* swarming cells is abolished by sub-lethal concentrations of antimicrobial compounds

Oihane Irazoki, Susana Campoy*, Jordi Barbé

*** Correspondence:** Corresponding Author: Susana.Campoy@uab.cat

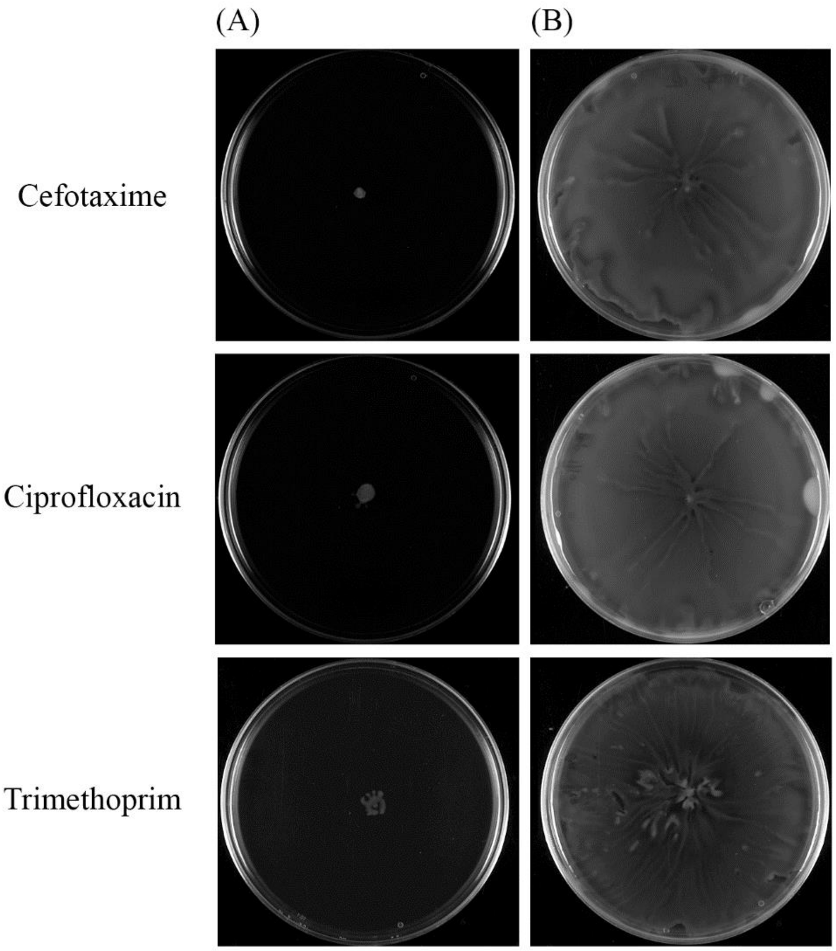

Supplementary Figure S3. Swarming ability of *S. enterica* cells treated with the sub-inhibitory and diluted sub-inhibitory concentrations of cefotaxime, ciprofloxacin and trimethoprim. The diluted sub-inhibitory concentrations were determined as the first one were no SOS response induction was observed. The no SOS system activation was confirmed by ELISA quantification of RecA. When diluted sub-inhibitory concentrations were used, the RecA amount within the cell was similar to that of non-treated cells while sub-inhibitory concentration treatments gave rise to at least 10-fold RecA increase. Diluted sub-inhibitory antibiotic concentrations were: 0.1 mg/L of trimethoprim, 0.053 mg/L of cefotaxime, and 0.00021 mg/L of ciprofloxacin.
