# Supplementary material for: The Transient Multidrug Resistance Phenotype of Salmonella enterica Swarming Cells Is Abolished by Sub-inhibitory Concentrations of Antimicrobial Compounds
Source: Front Microbiol. 2017 Jul 19;8:1360. doi: 10.3389/fmicb.2017.01360 (PMC5515874; doi:10.3389/fmicb.2017.01360)
Supplement: Supplementary file 4 [file Table_1.docx]

Supplementary Material

The transient multidrug resistance phenotype of *Salmonella enterica* swarming cells is abolished by sub-lethal concentrations of antimicrobial compounds

**Oihane Irazoki, Susana Campoy^*^, Jordi Barbé**

*** Correspondence:** Corresponding Author: [Susana.Campoy@uab.cat](mailto:Susana.Campoy@uab.cat)

**Supplementary Table S1.** Minimal inhibitory concentrations (MICs) and sub-lethal concentration of antibiotics for *S. enterica* ATCC14028 *ΔcheR* pUA1127 containing the *eYFP::cheR* fusion.

| **Antibiotics** | **MICs (mg/L)** | **Sub-lethal concentration (mg/L)** |
| --- | --- | --- |
| Amikacin | 16 | 4 |
| Cefotaxime | 12.5 | 1.6 |
| Chloramphenicol | 8 | 2 |
| Ciprofloxacin | 0.05 | 0.0065 |
| Colistin | 5 | 2.5 |
| Kanamycin | 10 | 5 |
| Tetracycline | 8 | 4 |
| Trimethoprim | 4 | 1 |
