# Supplementary material for: The Transient Multidrug Resistance Phenotype of Salmonella enterica Swarming Cells Is Abolished by Sub-inhibitory Concentrations of Antimicrobial Compounds
Source: Front Microbiol. 2017 Jul 19;8:1360. doi: 10.3389/fmicb.2017.01360 (PMC5515874; doi:10.3389/fmicb.2017.01360)
Supplement: Supplementary file 5 [file Table_2.PDF]

Supplementary Material

The transient multidrug resistance phenotype of *Salmonella enterica* swarming cells is abolished by sub-lethal concentrations of antimicrobial compounds

Oihane Irazoki, Susana Campoy*, Jordi Barbé

*** Correspondence:** Corresponding Author: Susana.Campoy@uab.cat

Supplementary Table S2. *S. enterica* ATCC14028 *ΔcheR* pUA1127 cell viability when treated with sub-inhibitory concentrations of the corresponding antimicrobial agent.

Antimicrobial	% Survival
Amikacin	79.01
Cefotaxime	69.14
Chloramphenicol	76.54
Ciprofloxacin	64.20
Colistin	74.07
Kanamycin	74.07
Tetracycline	66.67
Trimethoprim	64.20
